# Gastric Perforation After Liquid Nitrogen Cocktail Ingestion: A Case Report

**DOI:** 10.7759/cureus.82897

**Published:** 2025-04-24

**Authors:** Carlos I Zaldo Arredondo, Alfredo S Abarca Magallón, Roberto E Damacio Breton, Jose A Estrada Gonzales, Valeria Leal Isla Flores

**Affiliations:** 1 General Surgery, Hospital General de León, León, MEX; 2 General and Colorectal Surgery, Hospital Regional Lic. Adolfo López Mateos, Mexico City, MEX; 3 General Surgery, Hospital de Especialidades 5 de Mayo, Puebla, MEX; 4 Medicine, Benemérita Universidad Autónoma de Puebla, Puebla, MEX; 5 General Surgery, Cancun General Hospital "Jesus Kumate Rodriguez", Cancún, MEX; 6 General Surgery, Hospital Regional Lic. Adolfo López Mateos, Mexico City, MEX

**Keywords:** barotrauma-induced pneumoperitoneum, gastric perforation, liquid nitrogen, massive pneumoperitoneum, minimally invasive laparoscopy

## Abstract

Liquid nitrogen is a compound widely used in the food industry, and in recent years, it has gained popularity in the preparation of beverages and cocktails. However, it is not an innocuous substance. Although reported cases of gastrointestinal tract injuries associated with its ingestion are few, awareness of its potential harmful effects is crucial to reach a timely diagnosis and treatment. This article aims to present the case of a 34-year-old male patient who, following the consumption of a beverage prepared with liquid nitrogen, suffered a gastric perforation and massive pneumoperitoneum, as evidenced by a plain abdominal CT scan. He required emergency surgical intervention, which was successfully performed via a laparoscopic approach. The patient was discharged on the third postoperative day after tolerating a liquid diet.

## Introduction

Liquid nitrogen is a compound widely used in the food industry due to its ability to cool and preserve products through rapid freezing [1]. However, its recreational use in the preparation of food and beverages has also become popular, owing to the smoke effect it generates upon evaporation [2,3].

Inappropriate ingestion of liquid nitrogen can cause severe effects in the gastrointestinal tract, primarily due to direct contact with tissues, given its approximate temperature of -195°C (-319°F) [2,4]. Furthermore, its rapid expansion upon transitioning to a gaseous state can lead to gastrointestinal perforations and pneumoperitoneum secondary to barotrauma [4]. Although reported cases of digestive tract perforation associated with liquid nitrogen ingestion are rare, all have necessitated emergency surgical management [1-6].

Gastric perforation is suspected based on the patient's clinical presentation, which may include tachycardia, tachypnea, fever, generalized abdominal pain, and rebound tenderness as key symptoms [7]. The diagnosis is confirmed by evidence of free air in the abdominal cavity via imaging studies, among which computed tomography (CT) of the abdomen is the most useful [7,8]. Patients who undergo timely surgical intervention for gastric perforation tend to exhibit a favorable clinical course and can be discharged once they tolerate oral intake [7].

In the present report, we describe the case of a patient who presented with a gastric perforation associated with the recreational ingestion of liquid nitrogen, detailing the clinical evolution, surgical management, and subsequent postoperative course.

## Case presentation

A 34-year-old male patient, with no significant past medical history or prior surgical interventions, presented to the emergency department with acutely developing generalized abdominal pain after consuming a liquid nitrogen-infused alcoholic beverage at a bar.

Upon admission, the patient was diaphoretic and exhibited the following vital signs: tachycardia 124 beats per minute, blood pressure of 82/60 mmHg, a temperature of 35.4 °C, and a respiratory rate of 30 breaths per minute. On examination, he was lethargic, with notable generalized abdominal distension, rigidity, tender to palpation in all quadrants, tympanic to percussion, and demonstrated a loss of hepatic dullness. Relevant laboratory findings from the emergency department are summarized in Table 1.

**Table 1:** Laboratory test results

Test	Patient values	Normal range
Glucose	77 mg/dl	74 – 106 mg/dl
Urea	32 mg/dl	19 – 43 mg/dl
Creatinine	1.1 mg/dl	0.66 – 1.25 mg/dl
C-Reactive Protein	134 mg/l	≤5 mg/l
Leukocytes	21.46 x 10^3^/µL	4 – 10 x 10^3^/µL
Hemoglobin	16.60 g/dl	12 – 16 g/dl
Platelets	250 x 10^3^/µL	13-400 x 10^3^/µL
Sodium	139 mmol/L	137 – 145 mmol/L
Potassium	4.3 mmol/L	3.5 – 5.1 mmol/L
Chloride	108 mmol/L	98 – 107 mmol/L
Prothrombin Time	13.4 seconds	11 – 15 seconds
Partial Thromboplastin Time	29 seconds	26 – 36 seconds
International Normalized Ratio (INR)	1.03	

**Figure 1:**
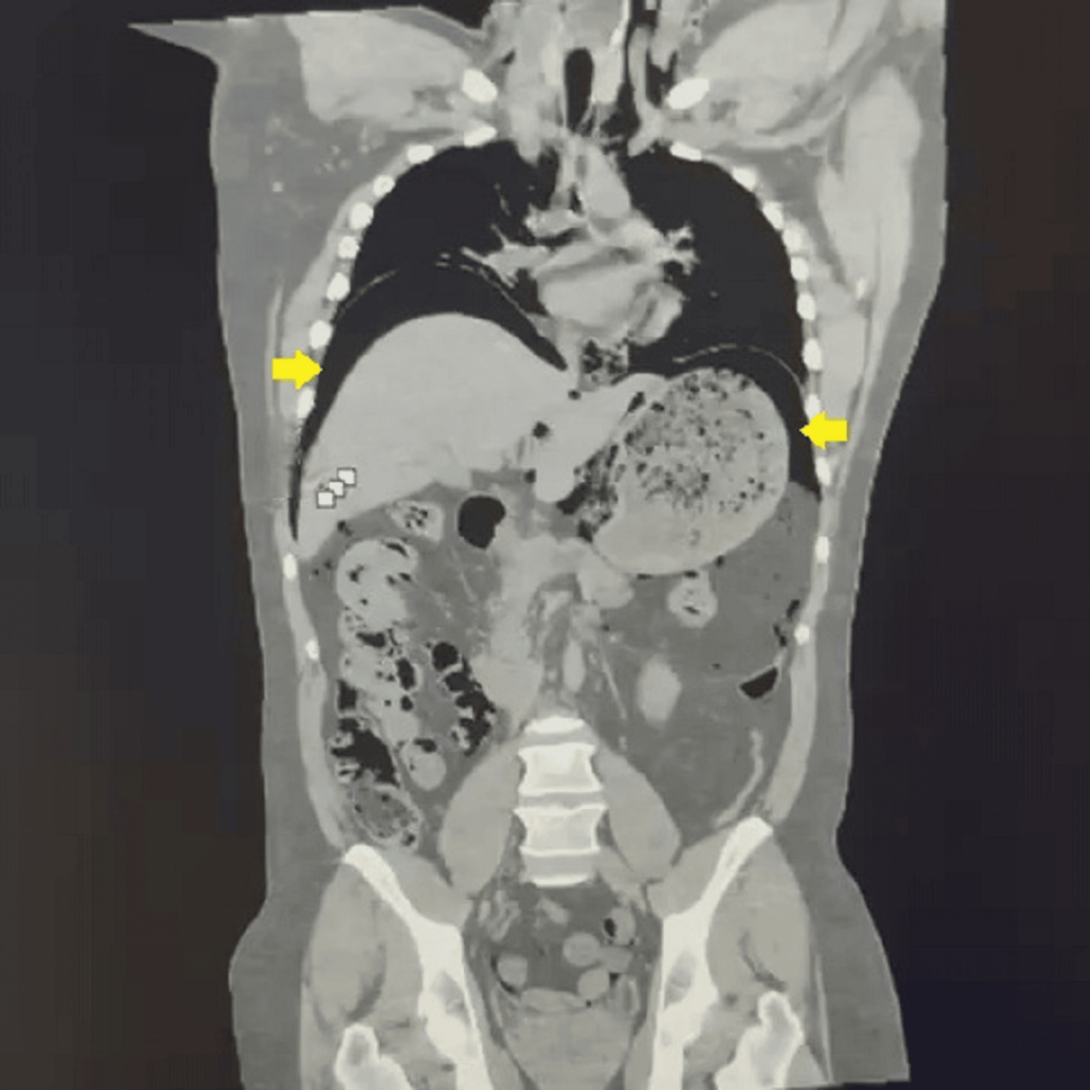
Abdominal CT scan showing pneumoperitoneum (yellow arrows)

Based on the clinical presentation and initial findings, an abdominal CT scan was immediately performed, revealing a massive pneumoperitoneum suggestive of hollow viscus perforation (Figure 1). This prompted a request for immediate diagnostic laparoscopy.

**Figure 2:**
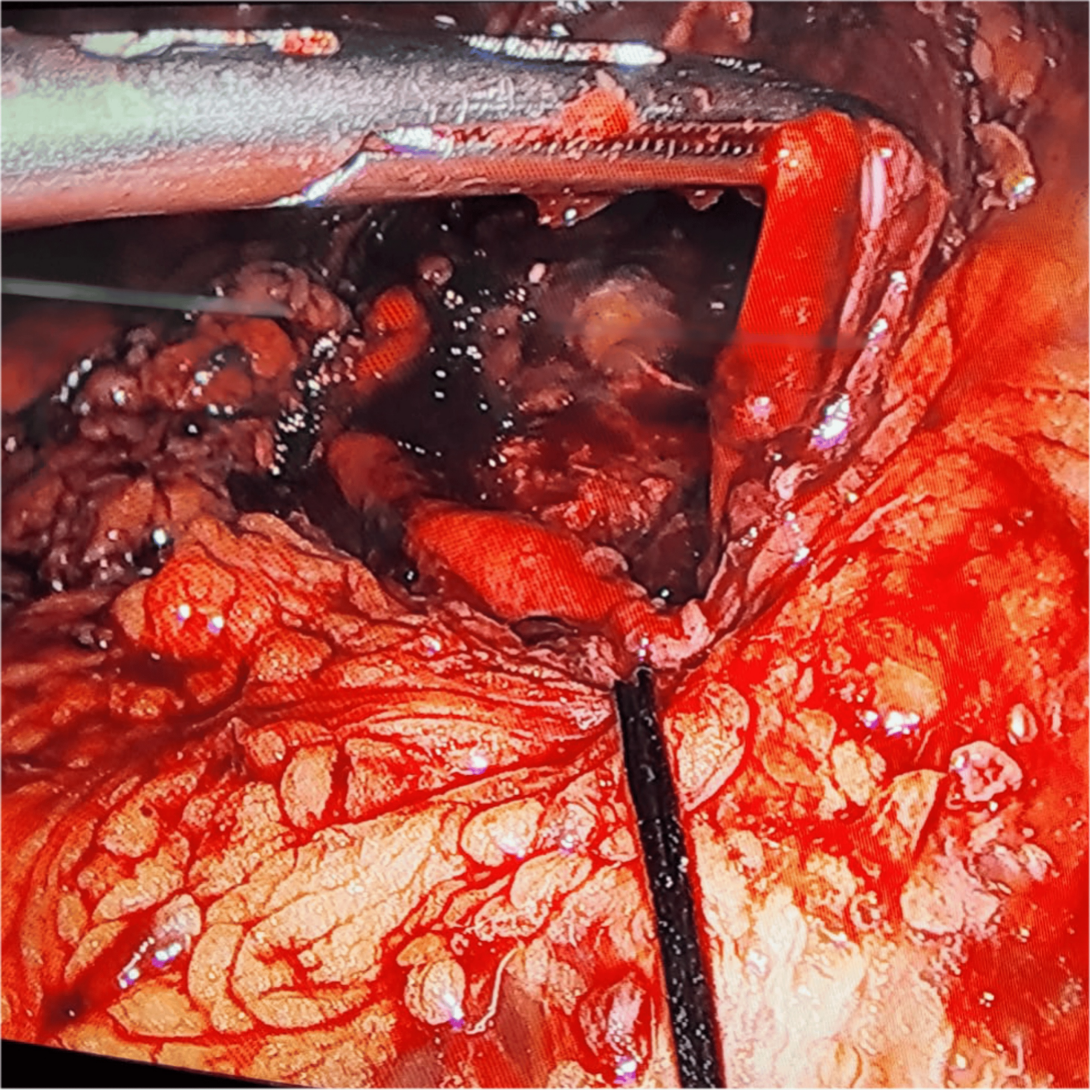
Exploratory laparoscopy revealing gastric perforation secondary to the ingestion of liquid nitrogen

**Figure 3:**
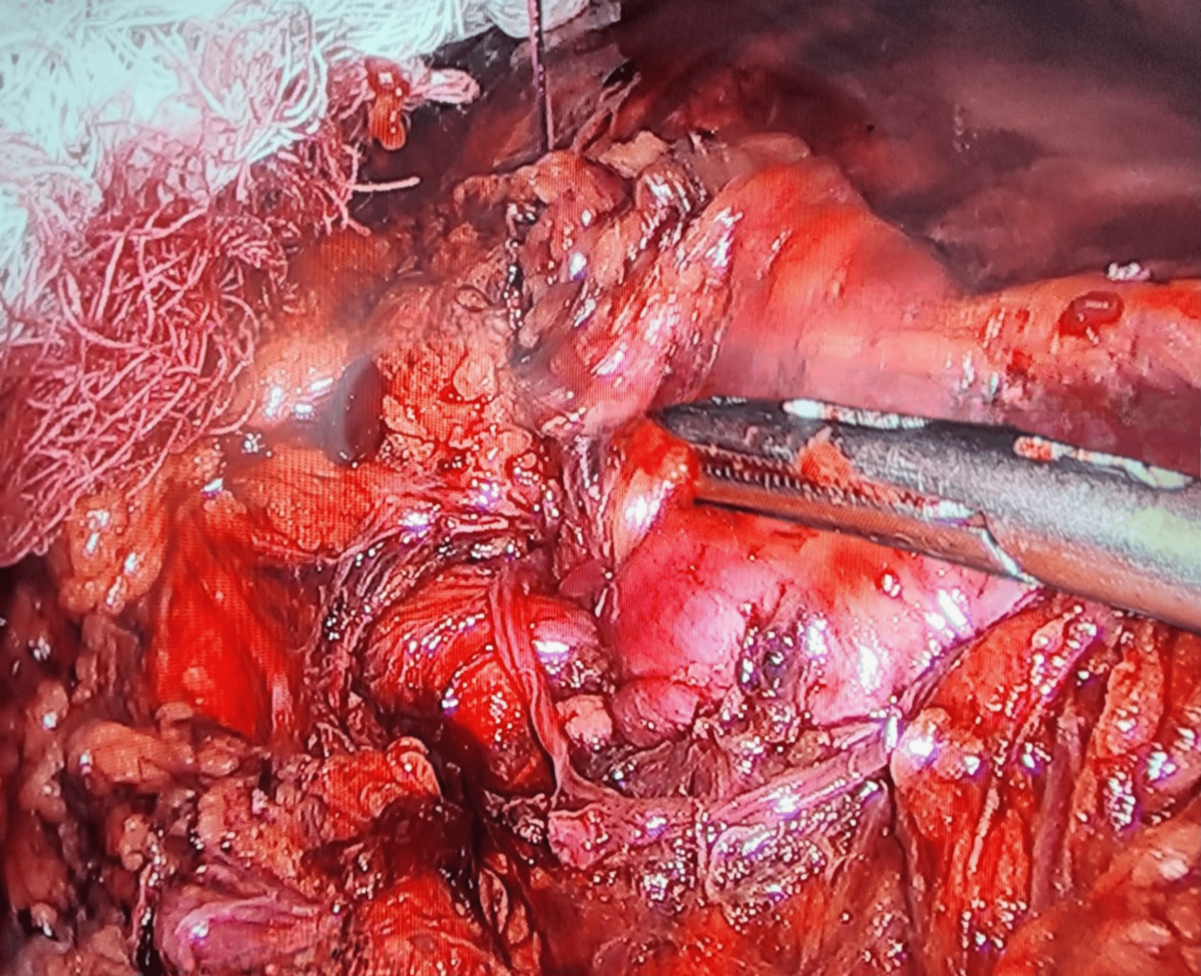
Repair of gastric perforation via laparoscopic approach with placement of an omental patch

Laparoscopic exploration revealed abundant free air in the abdominal cavity and a 3 cm perforation on the anterior aspect of the stomach (Figure 2), which was repaired laparoscopically with Monocryl 3-0 (Ethicon, Inc., Raritan, New Jersey, United States) and reinforced with an omental patch (Figure 3).

Postoperatively, the patient was transferred to recovery in stable condition and did not require mechanical ventilation. The patient progressed well, tolerating a liquid diet by postoperative day 2, and was discharged on postoperative day 3.

## Discussion

The etiology of gastric perforations can be diverse; however, the majority necessitate emergency surgical management due to the risk of peritonitis and abdominal sepsis [7,8]. Both clinical assessment and imaging studies are critical for timely diagnosis, with abdominal CT being the preferred modality due to its high sensitivity and specificity [7].

Surgical intervention can be performed via open surgery or laparoscopy, with the latter generally favored due to its minimally invasive nature. In most instances, management involves primary closure of the perforation site using absorbable suture material, with or without the application of an omental patch [7-9].

Reported cases of gastric perforation associated with liquid nitrogen ingestion are rare. These cases typically involve two primary mechanisms: cryogenic injury resulting from direct contact with mucous membranes and skin, and barotrauma occurring when liquid nitrogen transitions to a gaseous state within the gastric cavity [1-6].

The Leidenfrost effect describes the formation of an insulating vapor layer around a liquid when it encounters a surface with a temperature significantly exceeding its boiling point, thereby impeding heat transfer. This phenomenon explains how liquid nitrogen reaches the gastric cavity, where it undergoes rapid expansion as it vaporizes, with barotrauma serving as the primary mechanism of injury [4].

Irrespective of the etiology of gastric perforation and massive pneumoperitoneum, prompt diagnosis and surgical intervention are essential for achieving favorable patient outcomes [7,8,10]. Surgical management and subsequent follow-up do not differ significantly from other causes of gastric perforation [2,4-8]. The early initiation of enteral nutrition with good patient tolerance is a positive prognostic indicator for hospital discharge [7,8]. 

In this particular case, the minimally invasive laparoscopic approach, coupled with the accurate identification of pneumoperitoneum via CT imaging, played a pivotal role in the successful management of the patient.

## Conclusions

This report presents a case of gastric perforation resulting from liquid nitrogen ingestion. Prompt identification of the perforation was paramount in facilitating immediate surgical intervention, which led to the timely repair of the defect via a laparoscopic approach and a subsequent successful recovery for the patient. This case underscores the importance of raising awareness regarding the risks associated with the ingestion of cryogenic substances and emphasizes the critical role of healthcare professionals in recognizing atypical gastrointestinal injuries and cautioning the community about the possible damage that these can have on the digestive tract.
